# Popularizers, participation and the transformations of nineteenth-century publishing: From the 1860s to the 1880s

**DOI:** 10.1098/rsnr.2016.0029

**Published:** 2016-10-05

**Authors:** Bernard Lightman

**Affiliations:** 309 Bethune College, York University, 4700 Keele St, Toronto, Ontario, Canada M3J 1P3

**Keywords:** participation in science, popularizers of science, professionalization, scientific journals, the field and the laboratory, science communication

## Abstract

Focusing on the editors, journalists and authors who worked on the new ‘popular science’ periodicals and books from the 1860s to the 1880s, this piece will discuss how they conceived of their readers as co-participants in the creation of knowledge. The transformation of nineteenth-century publishing opened up opportunities for making science more accessible to a new polity of middle and working class readers. Editors, journalists and authors responded to the communications revolution, and the larger developments that accompanied it, by defining the exemplary scientist in opposition to the emerging conception of the professional scientist, by rejecting the notion that the laboratory was the sole legitimate site of scientific discovery and by experimenting with new ways of communicating scientific knowledge to their audience.

In the leadoff article for the first volume of their periodical the *Quarterly Journal of Science*, published in 1864, James Samuelson and William Crookes discussed the timeliness of their new venture.^1^ ‘We have been told by men in every walk of life’, they declared, ‘that the time is come when Science may claim for herself a special organ; that not alone scientific readers, but those of every class, desire to approach the source from whence this species of knowledge is derived … .’^2^ Samuelson and Crookes believed that there was a much broader audience for science in the 1860s that required journals, like their *Quarterly Journal of Science*, which appealed to non-specialists while cutting across class lines. Two years earlier, a journalist for *The Intellectual Observer* offered similar remarks in the first volume of the periodical in an article titled ‘The domestication of science’.^3^ Although ‘many families in the middle and upper classes are still deplorably ignorant of elementary laws and facts’, progress had been made in the last few decades. For ‘convincing proof’ that progress was real, the writer pointed to ‘the existence of our own magazine, and the wide welcome it has received in every town’. Tellingly, ‘[t]wenty years ago, no sane publisher would have ventured on such an experiment, and many who expressed their personal delight at our proceedings, thought we made a great mistake in offering matter of so high a class at so low a price’.^4^ Whereas earlier experiments in cheap scientific publishing designed to reach a wider audience had failed, the journal was a commercial success even though including original articles by skilled scientific authors was expensive. Those behind the journal were happy with the circulation of the new publication. They had not expected it to ‘circulate with the velocity of the penny novel or the startling romance’, but taking into account all of their readers they formed ‘a mighty host’.^5^

The *Quarterly Journal of Science* and *The Intellectual Observer* are examples of ‘popular science journals’ that were established in a period when the number of these types of periodicals increased dramatically (figure 1). According to Susan Sheets-Pyenson and Ruth Barton ‘popular science’ periodicals began to appear in the 1820s. Sheets-Pyenson estimates that there were nine popular science journals published in the 1820s. She includes three types of journals under the rubric of ‘popular science periodicals’: general popular science, popular natural history and mechanics’ magazines. From 1820 to about 1850 the total number of them remained virtually unchanged at 11 periodicals, but the numbers doubled during the 1850s and 1860s, peaking at 22 in the 1860s. After 1870 their production declined slightly.^6^ The growth of ‘popular science’ journals in Britain in this period was part of a larger development, what James Secord has referred to as the communications revolution: the outcome of new printing technologies, higher literacy rates, better systems of transportation and a reduction in the cost of paper as well as the lifting of the taxes on knowledge.^7^ Secord asserts that this revolution represented the ‘greatest transformation in human communication since the Renaissance’, which led to ‘opening the floodgates to a vastly increased reading public’.^8^

**Figure 1.**
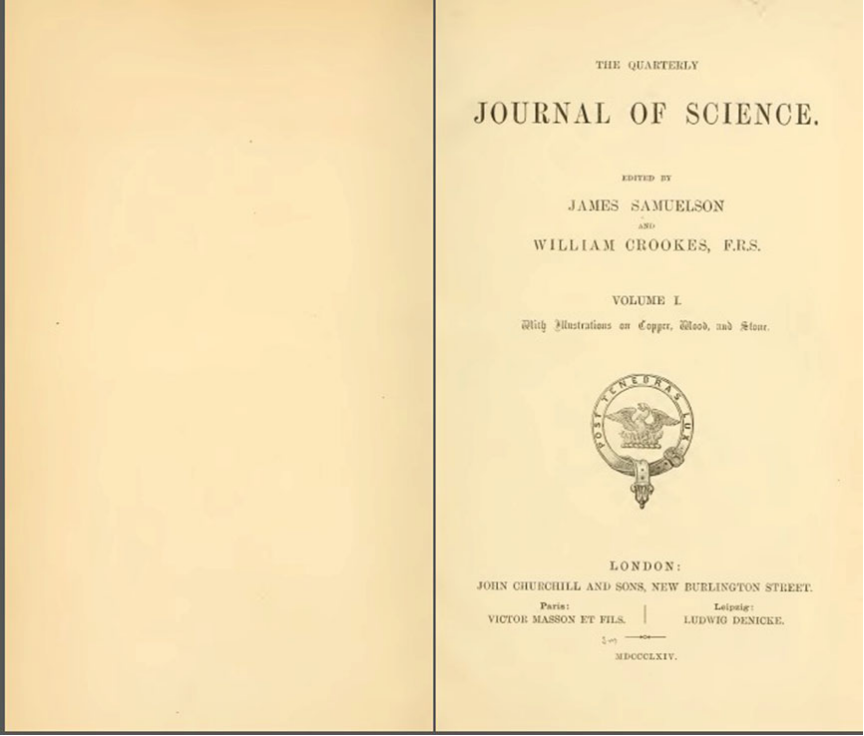
Title page of the first volume of the *Quarterly Journal of Science*. (Online version in colour.)

For British audiences this meant that the number of cheap, affordable science books and periodicals began to increase from about the 1820s on. As Jonathan Topham has shown, developments in the book trade in the 1810s and 1820s helped ‘foster and sustain new notions of popular publication in general and “popular science” publications in particular’. Topham argues that in 1815 the opportunities for most readers to have access to scientific reading material were ‘severely limited’. But the situation changed over the next 10 years when the production of cheap educational books for children and cheap journalism by small-time publishers helped to develop and define ‘popular’ publishing in general and especially ‘popular science’. The formation of the Society for the Diffusion of Useful Knowledge in 1826, as well as the founding of several cheap scientific periodicals, was a reaction to the commercial ‘popular science’ ventures of the earlier period. Now aware of a potentially untapped market, publishers were prepared to enter the field of self-consciously ‘popular’ publication.^9^ The publishing experiments in the 1830s, designed to explore this market, met with mixed success.^10^ But the spectacular sales of Robert Chambers's *Vestiges of the Natural History of Creation* (1844) ushered in a new era of science publishing. The success of Routledge's series of shilling handbooks on natural history begun in the late 1850s encouraged publishers to set up their own scientific series in the 1860s and the 1870s. Titled the ‘Common Objects’ series, the books focused on familiar things that could be found at the seashore or in the country.^11^

The 1860s, 1870s and 1880s differed profoundly from earlier periods. First, the poor showing of British manufacturers at the Paris International Exhibition in 1867 had reignited a lively debate—which had also erupted in the 1830s and 1840s—on the quality of technical instruction in Britain. Scientific lobbyists such as Lyon Playfair asserted that to avoid lagging behind the rest of Europe required the scientific training of the manufacturing population. This led to a demand for teachers trained in natural science in schools run by the Department of Science and Art to serve the industrial population. Second, the Reform Act of 1867, which gave the working class the vote, made it clear that universal education was needed to produce a more informed electorate. Third, the government passed the Education Act in 1870, a response to the perceived need to educate the workers. In those areas of the country where the churches and other voluntary organizations had not provided enough schools, the Act granted locally elected school boards the power to establish schools and to levy a rate to pay for them. All of these developments stimulated the need for more cheap science textbooks for the schools and for the production of more ‘popular science’ works for the entire population.^12^ At the same time, the composition of the scientific community was undergoing significant change. For reformers like T. H. Huxley, who had arrived on the London scientific scene in the early 1850s, it was imperative to redefine science so that it was associated more closely with expertise, laboratory research and naturalistic methods. He wanted to break its connection with the Anglican clergy and natural theology. For Huxley and his allies this meant pushing those who did not agree with their vision of science out of scientific societies. Many of the fierce debates that took place over such controversial issues as evolutionary theory were, at a deeper level, also about the nature of the scientific community and who should have the authority to speak on behalf of science. This does not mean that the process of professionalization was complete in the second half of the century; only that some of the characteristics of modern professional science were desired by nineteenth-century figures like Huxley.^13^

Focusing on the editors, journalists and authors who worked on the new ‘popular science’ periodicals and books from the 1860s to the 1880s, this piece will discuss how they conceived of their readers as co-participants in the creation of knowledge. The transformation of nineteenth-century publishing opened up opportunities for making science more accessible to a new polity of middle and working class readers. Editors, journalists and authors responded to the communications revolution, and the larger developments that accompanied it, by defining the exemplary scientist in opposition to the emerging conception of the professional scientist, by rejecting the notion that the laboratory was the sole legitimate site of scientific discovery, and by experimenting with new ways of communicating scientific knowledge to their audience.

## The exemplary scientist

Natural historian and popularizer Frank Buckland, son of the better-known Oxford geologist William Buckland, was adamant that there should be no bar to participation in science. Buckland was an influential popularizer of science. Much in demand as a lecturer, he also wrote an endless stream of contributions to periodicals and authored six books. His *Curiosities of Natural History*, first published in 1857, went through several series.^14^ Buckland believed that it was important to write scientific books that were accessible to the general reader. Instead of communicating knowledge by using ‘hard words and unnecessary mystifications’, Buckland wished, ‘on the contrary, to throw the portals of Science, (i.e. knowledge,) wide open, and let all enter who will’.^15^ Moreover, he believed that ‘an immense amount of valuable practical knowledge lies unutilized in the minds of those of the humbler classes who have neither the ability nor the opportunity of expressing it in writing or in print’.^16^ To illustrate his point, Buckland wrote about his conversation with a retired, one-armed sailor. Although his answers to Buckland's questions were rendered in a rough working class dialect, the sailor had valuable information about sea snakes, big cuttlefish and whales. In this essay the supposed expert, Buckland, was educated by a semi-articulate mariner.^17^ Throughout his books on natural history, Buckland introduced zookeepers, rat catchers, fishermen and others who had practical knowledge of animals since they were, so to speak, constantly in the field doing their research. They were the ‘amateurs’ that would-be professional scientists, like Huxley, wanted to exclude from scientific societies and from science itself. But Buckland was expressing a sentiment widely shared among popularizers. As Sheets-Pyenson and Barton have asserted, a participatory, republican image of scientific community was important for popularizers from the early nineteenth century to at least the 1860s.^18^ I would argue that for many popularizers the republican scientific ideal continued to be influential into the 1880s.

Exemplary scientists were depicted by popularizers in books and periodicals in accordance with the participatory ideal.^19^ By ‘exemplary’ I mean highly regarded scientific figures that were praised as worthy examples that could be imitated by any member of the public, no matter the nature of their social background or the extent of their training. John Henry Pepper, popularizer and manager of the Royal Polytechnic Institution, offered a biographical account of eminent natural philosopher Michael Faraday in his *Cyclopaedic Science Simplified* (1869), which stressed that his humble beginnings did not prevent him from making ‘a name that few have or will ever be able to achieve’.^20^ The self-educated and working class Faraday was a model that readers could imitate. But even important scientific figures who came from a wealthy family and who had received formal scientific training could be presented in a similar light. Alexander von Humboldt was portrayed in a biographical study in the journal *Recreative Science*^21^ as a model of perseverance that demonstrated how it was possible to rise in the world.^22^

But accounts of Charles Darwin are perhaps the most intriguing example of how a significant scientist was treated as an inspiration to those who were self-educated and not among the wealthy. Narratives about Darwin in the nineteenth century varied dramatically. Many popularizers were fond of discussing him as if he were a natural historian, not a professional biologist. Robert Ball, Lowndean Professor of Astronomy and Geometry at Cambridge University, and a prolific popularizer of astronomy, referred to Darwin in 1892 as ‘the Newton of natural history’.^23^ The Spencerian popularizer, Grant Allen, pointed out that Darwin ‘was not a professor. He was not a trained physiologist. He was not a drilled and dragooned South Kensington student’. Rather, Darwin was ‘merely an amateur, a lover of truth, who was impelled by curiosity’.^24^ Finally, popularizer Eliza Brightwen offered Darwin as an example for her readers of what could be accomplished by anyone who studied the minutest objects in nature. Brightwen looked ‘with wonder at the work of such a student as Darwin, who gave twenty long years to observation of the common earth-worm before he wrote his deeply interesting book upon it’.^25^ Richard Proctor, a popularizer of astronomy who founded the journal *Knowledge* in 1881 to challenge what *Nature* stood for, was militantly anti-professional.^26^ He criticized ‘*soi-disant* professional astronomers’ who treated the wonders of the heavens as ‘a mere land surveyor might discuss the teachings of the earth's crust’.^27^ But Darwin he regarded as one ‘who had done more than any since Newton to extend men's recognition of the wideness of the domain of law’.^28^

Proctor himself was celebrated in the pages of journals popularizing science. Although Proctor would not seem to be in the same category as Faraday, Humboldt and Darwin, he was well known to the Victorian reading public. Author of over 60 books during his career—perhaps the most of any nineteenth-century popularizer—he was a prolific contributor to a host of scientific periodicals. He also lectured extensively around the world, including tours of the United States, Canada, Australia and New Zealand.^29^ Moreover, Proctor had established his credentials as an astronomer by submitting technical articles to the *Monthly Notices of the Royal Astronomical Society*, by becoming editor of the *Monthly Notices* in 1872 (though only for a brief time) and by obtaining appointment as an Honorary Secretary of the Royal Society. He steadily increased his popularizing activities after 1873. To the Victorian reader, Proctor was as much an exemplary scientist as Faraday, Humboldt or Darwin. *Scientific Opinion* reviewed his book *Other Worlds Than Ours* (1870), praising his skill in conveying knowledge while adding ‘to our stock of scientific information’.^30^ The journal was equally enthusiastic when it reported on Proctor's lectures on star-drift and nebulae at the Royal Institution.^31^ ‘Once more’, the reviewer for *Scientific Opinion* declared, ‘Mr. Proctor has triumphed, not only as an original observer of persevering enterprise and surprising acuteness, but as one of the best popular teachers of astronomy which the country has produced.’^32^

At the same time *Scientific Opinion* could be critical of professional scientists who were too speculative for its taste. John Tyndall was taken to task for his claims about the existence of organic matter in dust. A report on a Royal Institution lecture delivered by Tyndall on 21 January 1870, on haze and dust, drew a sharply worded rebuke from the *Scientific Opinion* writer. Although Tyndall's demonstrations were usually convincing, the writer complained that on this occasion ‘he formulated such grave propositions, and to our minds supported them so imperfectly, that we must own to leaving the lecture theatre in a very sceptical frame of mind’. *Scientific Opinion* rejected Tyndall's assertions that the air we breathe is packed with minute organic particles that are germs of plants like yeast and other such fungus, and that they were responsible for epidemics. Tyndall had not offered sufficient proofs for these controversial statements.^33^ Since Tyndall's lecture was, to him, an important statement of his position on the spontaneous generation debate, and since his reputation as a trusted experimenter was being questioned, he felt compelled to reply.^34^ That he decided to do so was an acknowledgement of the influence of *Scientific Opinion*. Although Tyndall responded to the negative review of his lecture, *Scientific Opinion* published articles in subsequent issues that provided more evidence against his position, including an article taken from *The Lancet* that concluded that the eminent physicist had ‘accomplished nothing but the generation of distrust in his own sagacity’.^35^

In her recently published *Making* Nature: *the history of a scientific journal*, Melinda Baldwin asserts that at times *Nature* ‘served as a site where its contributors could explore what it meant to be a scientist’.^36^ The same can be said about books and periodicals devoted to popularizing science. But their conclusions diverged significantly from those reached by contributors to *Nature*, which, after it was first founded, rapidly became a journal for would-be professionals. Through their books and periodicals, popularizers tried to define the term ‘scientist’ in a way that included a much broader spectrum of scientific workers. They applauded the work of exemplary scientific figures such as Faraday, Humboldt, Darwin and Proctor. Instead of emphasizing that their great achievements were the result of professional training, popularizers pointed to their lowly origins, their powers of observation or their love of truth. The lesson for readers was obvious. They, too, could participate in the pursuit of scientific knowledge. They also had the abilities to make great discoveries. They were not limited to subordinate roles, such as collecting specimens for the professional scientist. Discovering knowledge was not reserved solely for the trained expert. Indeed, professional scientists like Tyndall could easily fall into error when they indulged in speculation.

## Sites of knowledge

Proctor's disdain for professional scientists can also be seen in his sarcastic remarks about scientific societies. Shortly after the British Association for the Advancement of Science meeting at Southampton in 1882 he asked in the pages of *Knowledge* if the work done by the Association during the 50 years of its existence was really ‘worth the time and labour bestowed on the meetings’? He was especially critical of the jargon used in the presidential addresses, which could not be understood by the vast majority of the audience, and of reports of these addresses in such periodicals as *The Times* and the *English Mechanic*, which merely repeated ‘this egregious nonsense, as if it had been profound science’.^37^ Supposedly offered for the general public, the presidential address was nothing but a bore. The problem with the presidential address raised larger questions about the effectiveness of the British Association as a society devoted to disseminating science.

But Proctor reserved his harshest criticisms for the elite Royal Astronomical Society, charging that it was dominated by a small clique of professional astronomers who delivered tedious papers at meetings, published their own observations in official Society publications and used the Society merely to further their own careers. Proctor was outraged by the abuses he saw in award competitions and elections to Society offices. The process whereby medals were awarded ‘very readily lends itself to jobbery’, while the presidency was seen by the members as a stepping-stone to a salaried post.^38^ Proctor maintained that scientific societies tended to discourage rather than advance science. ‘And I am confirmed in this belief by noting’, he added, ‘that not one series of scientific researches of any importance has attained success through the influence of any scientific body, or has ever been materially helped by such influence.’^39^ In essence, Proctor was insinuating that scientific societies were not set up to foster the growth of science, but rather to promote the selfish interests of their members. Like other popularizers, Proctor was hostile to the entire concept of a scientific establishment. Proctor's views were somewhat extreme by the 1880s, because by then, as Sheets-Pyenson asserts, a younger generation of scientists had for two decades being trying ‘to mould the Republic of Science's amateur practitioners into sympathetic supporters of professional high science’, despite resistance from avid amateur naturalists.^40^ However Proctor's remarks were generally indicative of how some popularizers rejected the notion that science could be practised by professionals only in a small number of spaces, such as laboratories or scientific societies.

The laboratory became the privileged site for aspiring professional scientists in the second half of the nineteenth century, thanks, in part, to the efforts of men like Huxley. It was Huxley who argued that laboratory work had to be an integral component of the training of biology teachers in England so that they could teach proper experimental methods to their students. One of his central goals as a member of the Devonshire Commission (which met from 1870 to 1874) was to establish the laboratory as the privileged site of the scientific discovery as well as the best space in the schools and universities for students to learn in.^41^ Huxley championed the laboratory when, owing to what Kohler and Vetter refer to as the laboratory revolution of the 1840s to the 1880s, it became the place where high-status, modern science was carried out. The spatial and epistemic reorganization of science that followed cast ‘the field’ in the role of ‘the not quite modern kind of science associated with amateur field activities that were not properly quantitative, experimental, hypothetico-deductive, and analytically rigorous’.^42^

By contrast, popularizers believed that scientific work could be done almost anywhere and that members of the public could participate in the making of knowledge in different ways. This, of course, was connected to the desire to keep science accessible. Popularizers of science stressed the importance of studying common, everyday objects that were easily within the reach of everyone. In the midst of the laboratory revolution the popularizers were extolling the virtues of field science, arguing that the field was just as important as a site of scientific research as the laboratory. In the article on ‘Common things’, a writer for *Science Gossip* criticized those amateurs who thought that the scientific life consisted of collecting rare plants and animals.^43^ ‘It is not the *rare* but the *common* species which give character to a flora or fauna’, the author insisted, ‘and the time spent in hunting after, or travelling for miles in pursuit of some rarity, would be better employed in cultivating a closer acquaintance with such “common things” as “buttercups and daisies”, or ladybirds and “cabbage-whites”.’ The true naturalist studied the life history of the honeybee, the earthworm or other common things.^44^ *Science Gossip* was not alone in its emphasis on common things. The popularizer of geology, David Page, for example, insisted that the objects of study were scattered everywhere around us, in quarries, railway cuttings, glens and mountains.^45^ Brightwen advised her readers that the study of plants could be ‘carried on almost everywhere’, while John George Wood, among the most widely read popularizers of natural history, pointed to the ‘wonderful amount of interest which lies hidden in every object around us’. There was ‘no need of travelling to tropical countries for Nature's marvels’, Wood declared.^46^ Darwin, Wood implied, could just as well have stayed in his own backyard to make important scientific discoveries. In his *Common Objects of the Microscope* (1861), Wood actually referred to the ‘many most curious and valuable original observations’ made ‘by an old lady in her daily perambulation to a little scrap of a back yard in the suburbs of London, barely twelve yards long by four wide’.^47^

Popularizers wrote about the discoveries to be made in the most common, mundane places. In the pages of the journal *Recreative Science*, Tuffen West, known as a scientific illustrator as well as a popular science writer, discussed the ‘Wonders of a stagnant pool’. While on a walk near his home, West collected various plant specimens from a stagnant pool of water that provided ‘material enough for some hours’ careful examination, and, if time permitted, months, it may be years, of study’.^48^ Using a microscope, he found fascinating minute plants in the material he had collected; but they were puzzling. ‘How instructive the little we yet know about them, and how much of yet greater interest remains to be learnt of the history of their life’, he exclaimed. However, West was convinced that they played a significant role in the economy of nature. Though seen by the ‘popular mind’ as pernicious, these miniscule plants fed on ‘agencies destructive to our life’ while oxygenating the water.^49^ In the end, they served an important purpose.

Similarly, Buckland wrote a long essay exploring what could be found in a horse-pond. Buckland begins by anticipating the question that will be asked by the incredulous reader: ‘Pray what is there to be found in a horse-pond except mud, dead dogs and cats, and duck-weed?’ Buckland answers that the thoughtful natural historian would find life ‘in all diversity of form, beautifully and wonderfully arranged’.^50^ All of nature is worthy of study because life could be found everywhere. Buckland then studies the horse-pond for signs of life. He invites the reader to ‘choose a dry place at the side, and fix our eyes steadily upon the dirty water: what shall we see?’.^51^ Gradually, by patiently observing, the ‘pond-world’ reveals its secrets. The emergence of a frog's head from the water leads Buckland to discuss what frogs eat, what they taste like if eaten, their noises, how they hibernate and some of his previous experiences with these creatures. This leads him to recollect encounters with toads, lizards, shrimp, lobsters and even the remains of dinosaurs. Buckland treats them as if they are related in different ways to the frog. The horse-pond serves as a familiar jumping-off point for Buckland to examine a series of animals that are all connected to the ecology of the humble ‘pond-world’.

Besides stressing the importance of common, mundane places in nature, popularizers could also insist that public spaces of science, such as aquaria and museums, were perfectly legitimate sites in which to undertake scientific investigation. In these sites, members of the public could also participate in the making of scientific knowledge. Whereas original research could be done in the mundane places of nature, members of the public could witness scientific discovery in action in aquaria and museums. Buckland referred to the Brighton and Southport aquaria as ‘great educational schools’.^52^ After 1867, when Buckland had been appointed Inspector of Fisheries, his thoughts on the importance of aquaria would have carried significant weight.^53^ The Brighton Aquarium, Buckland declared, would ‘not only enable us to make great advances in the knowledge of marine fauna, but will also much assist in solving difficult fishery questions relating to the management of fisheries. If the Brighton Aquarium does not turn out to be a key which will unlock many difficulties in practical history, I shall be much disappointed’. To Buckland, the Brighton Aquarium, like other aquaria, was really a ‘marine observatory’ that could be used by scientists to discover the ‘secrets of nature’ while also educating the public.^54^ Whereas scientists often conceived of the laboratory as a restricted space for professionals or professionals in training, Buckland saw aquaria as open sites where members of the public could witness the discovery of new knowledge.

**Figure 2.**
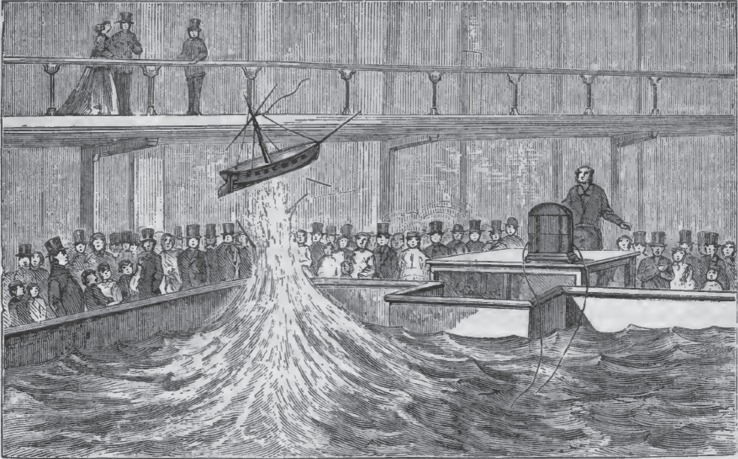
Pepper's miniature torpedo experiment. (From J. H. Pepper, *Cyclopaediac Science Simplified* (Frederick Warne & Company, London, 1869), p. 344.

Similarly, Pepper drew attention to his own Royal Polytechnic Institution as a scientific site of importance. In his *Cyclopaedic Science Simplified* (1869), Pepper referred to ‘the miniature torpedo experiment performed so frequently at the Royal Polytechnic’. A copper cylinder containing a few grains of gunpowder was sunk two feet in the centre of a large tank filled with water. When a spark was passed to the copper cylinder through wires attached to it, it exploded, blowing a model ship high into the air.^55^ By presenting the demonstration as a repeatable experiment, Pepper was supporting his larger claim that legitimate scientific research was being done at his polytechnic (figure 2). *Scientific Opinion* took Pepper's claim seriously by reproducing a lecture by Benjamin W. Richardson that had been reported in the *Medical Times and Gazette*. In the lecture, Richardson, well known for his research into anaesthetics and for his work in public health and the sanitary movement, described the medical research he had conducted with the polytechnic's large induction coil.^56^ Richardson recalled seeing the ‘splendid induction coil’ in action at the polytechnic, where it provided ‘for the scientific instruction of the people’. It occurred to him that the instrument, ‘the most powerful electrical instrument of its kind ever constructed’, could be used ‘on behalf of medical science’ by settling ‘some doubtful points of great importance’ still unresolved. One of those points concerned ‘the phenomena attendant upon death by lightning-stroke’.^57^

Popularizers of science insisted that scientific investigations could be done almost anywhere, and not just in the privileged sites of professional science. Science consisted in the study of common objects that were within the easy reach of all. These objects could be found in the most mundane, everyday places, such as stagnant pools, horse-ponds or even one's own backyard. Public scientific spaces, such as the Brighton Aquarium or the Royal Polytechnic Institution, were also valid sites for undertaking research and for the witnessing of new discoveries. They were not merely sites of science popularization in which visitors passively consumed old scientific truths already uncovered by professional scientists. By expanding the number of legitimate sites in which scientific research could be undertaken, popularizers of science maintained that diverse sites offered different levels of participation to members of the public. Participation in the making of science did not require gaining access to a laboratory or travelling to exotic locations to obtain rare specimens.

## Communicating knowledge

Just as popularizers conceived of the exemplary scientist and the spaces in which they worked in a way that made science more widely accessible, they also discussed how to communicate knowledge in a manner that invited broad participation. In his *Illustrated Natural History* (1853), Wood declared that he was going to present the outlines of ‘zoologic knowledge’ in a ‘simple and readable form’ by removing ‘the technical language of scientific zoology’. The true object of zoology was not to ‘arrange, to number, and to ticket animals in a formal inventory, but to make the study an inquiry into the Life-nature, and not only an investigation of the lifeless organism’.^58^ In other words, communicating knowledge required non-exclusive language and formats that brought the subject alive. Since Wood's book was heavily illustrated, as were many of his works, he would have added that communicating knowledge was best accomplished by using pictures as well as words.

**Figure 3.**
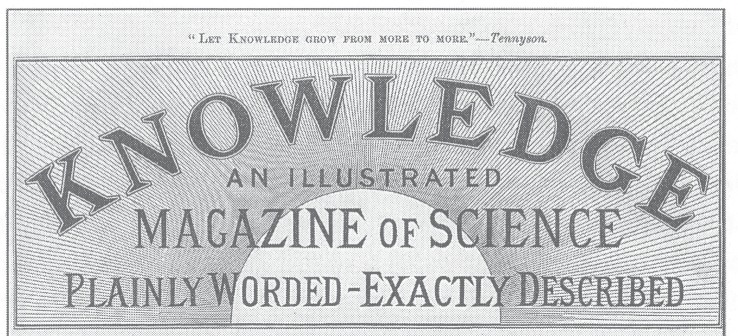
The masthead of Proctor's journal, *Knowledge*.

**Figure 4.**
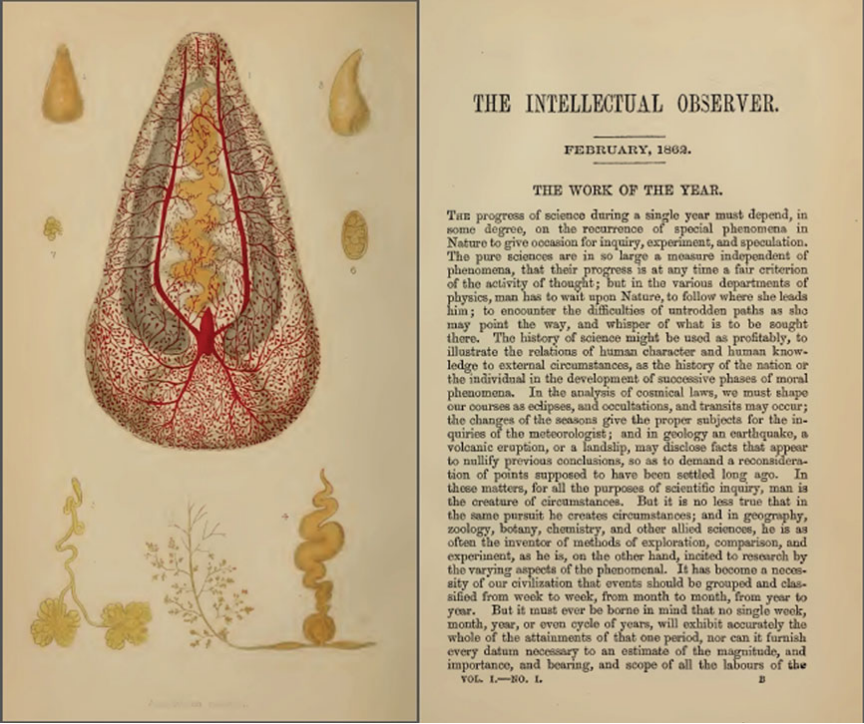
A colour plate from *The Intellectual Observer*. (From *Intellect. Obs.* **1**, [vi] (1862).) (Online version in colour.)

Alice Bodington, who popularized evolution and biology in the final decades of the century, insisted that knowledge ‘must be made clear and plain to the many’. She praised Darwin for having the ‘extraordinary genius and patience’ to make ‘original observations’ while also making ‘the result of his investigations clear and plain to any person of ordinary intelligence’.^59^ Popular science journals adopted a similar plan. In his opening editorial statement to the readers of *Knowledge*, Proctor declared that his weekly magazine was ‘intended to bring the truths, discoveries, and inventions of Science before the public in simple but correct terms’.^60^ The subtitle of Proctor's journal was ‘An Illustrated Magazine of Science Plainly Worded—Exactly Described’ (figure 3). The fact that it was illustrated points to another common feature of popular science books and journals. Some even included expensive colour plates, such as the first volume of *The Intellectual Observer*, which featured six colour illustrations (figure 4). Illustrations and clear language were often used to convey the connectedness of nature and unity of science. In their statement of ‘The Endeavour’ they had undertaken, the editors of *Recreative Science* made the ‘linking of departments of knowledge together by the threads of their inevitable connection’ as one of their primary goals.^61^ Page argued that there could be no true knowledge of any one branch of natural science without some acquaintance with the whole.^62^ Opposed to the fragmentation of science, he was responding to the proliferation of specialist journals as well as specialization in general. Commercial science periodicals, which often focused on a narrowly defined subject, had grown from five journals in 1815 to over 80 by 1895.^63^

Proctor asserted that it was difficult to find writers for his journal who knew how to communicate with a general audience. Specialists were more likely to provide a narrow view of their subject in addition to using technical words. ‘I cannot get from such men the kind of writing I want’, he complained.^64^ As a result, Proctor tended to write a large number of the articles for *Knowledge* himself. Other popularizers recognized Proctor's gift for writing clearly and accessibly for the Victorian reader. In a review of Proctor's *Other Worlds Than Ours*, the book was praised for ‘treating learnedly, and yet simply and intelligibly, of those great questions concerning the laws of the universe, of which the outside world has lately heard so much and understood so little, and it is written in such excellent English that its literary merits are nearly equal to its scientific ones’.^65^

Many editors of popular science journals believed that to reach the new audience for science it was necessary to think in creative ways about how to format their periodical, in addition to using clear, non-technical language, more illustrations and taking a synthetic approach to scientific topics. The journal *Knowledge* provides an excellent example. Through his extensive use of correspondence and gossip columns Proctor had intended to establish a weekly that championed the ‘Republic of Science’ tradition of earlier science journals for general readers.^66^ He repeatedly changed the format of the journal in order to find the right formula for success. In 1885 he decided to take the radical step of publishing *Knowledge* as a monthly. He acknowledged that ‘*Knowledge* as a monthly magazine must be an experiment’.^67^ Proctor claimed that he was willing to dispense with the customary role of editor. He chose to ‘talk thus familiarly with his readers’ instead of standing on a ‘pedestal on which to assume statuesque editorial dignity’.^68^

The editor of *Scientific Opinion*, Henry Lawson, also thought that a correspondence section was important for a popular science journal. In reviewing the initial volumes of the journal, the author of ‘Our Past and Our Future’ did not believe that the correspondence section required any defence. ‘The correspondence speaks for itself’, the journalist insisted. ‘We have had occasionally to admit letters we should rather have rejected; but believing that even in many cases, where unquestionable rubbish has been written, there were a few grains of truth and sense, we have preferred preserving the grain to destroying the tares and corn together.’ *Scientific Opinion* attempted to keep the correspondence section open to all. Side by side with the letters containing ‘unquestionable rubbish’ there were also communications ‘from Darwin, Gosse, Wallace, Proctor, Sterry Hunt, David Forbes, Church, and others’, containing matters ‘of the utmost interest to men of science’.^69^ All correspondents, including those who were well-known men of science, were presented as members of a single community of equals, despite the occasional letter filled with nonsense.

The communications revolution had raised central questions about how science should be conveyed to the growing popular audience. Popularizers valued accessibility, clarity and simplicity. They appreciated the appeal of visual images. They often used language and images to point to the connectedness of nature reflected in the unity of science. By contrast, in the opinion of some editors, specialist experts were too narrow and technical in their writing to reach the general reader. W. T. Stead, for example, editor and proponent of the ‘new journalism’, recommended that newspaper editors ‘never employ an expert to write a popular article on his own subject’. It was far better to employ ‘someone who knows nothing about it to tap the expert's brains, and write the article, sending the proof to the expert to correct’. The problem with experts was that they forgot that they were ‘not writing for experts but for the public, and will assume that they need not be told things which, although familiar to him as ABC, are nevertheless totally unknown to the general reader’.^70^ From the 1860s to the 1880s many popular science journal editors encouraged broader participation by their audiences through the use of innovative formats and more extensive use of correspondence columns.

## Reflections

In the introduction to the first issue of his new journal, the *Popular Science Review*, published in 1862, James Samuelson offered an example that symbolized scientific progress. He pointed to how knowledge had been pursued in ‘former times’, when ‘representatives of science’ secluded themselves from society as they pored over philosophical works. Passing day and night engaged in experimenting upon the elements, they sought to convert the baser metals into gold. The alchemists never found what they were searching for; but modern science had made discoveries that were even more stunning—and magical—than the secret of transmuting base metals into gold, such as the invention of the steam engine or anaesthetics. Unlike the alchemist, and this was the key point for Samuelson, modern scientists did not keep their discoveries a secret. The spread of knowledge required the ‘united efforts of many minds’. As a result, ‘the men whose avocation it is to penetrate nature's secrets, do not now, as formerly, work alone, secluded from the world, and surrounded by mysteries impenetrable to the vulgar gaze, as were their ancestors’. Instead, they competed ‘with one another in imparting, not in concealing, information’. The hallmark of modern science, then, was that the ‘thoughtful sage’, who spent his nights in private study, rambled ‘through country lanes’ during the day, ‘surrounded by anxious inquirers—youths and maidens, the aged and young—all anxious to secure a little of the knowledge which he has secured at the cost of so much toil, but now dispenses with such a liberal hand’.^71^ To Samuelson, modern scientists openly communicated the knowledge they discovered to an interested public. Those scientists who thought that professionalization meant that the public should be excluded were, by implication, analogous to the secretive alchemist.

Crucial questions were still open to debate during the period from the 1860s to the 1880s, questions such as: What is science? What is a scientist? Who can participate in the making of knowledge? Popularizers argued that science should be accessible to all, communicated through clear language and visual images rather than through jargon. They also contended for a broader definition of the term ‘scientist’ that was not restricted to those with specialized training or expertise. Members of the public could actively participate in the pursuit of scientific truth in different ways, not just as passive consumers of elite science or as collectors of specimens or data for would-be professional scientists working in the laboratory. In the public spaces of science, such as museums and aquaria, they were legitimate witnesses to important experiments. In the field, they conducted research that could potentially lead to ground-breaking new scientific discoveries like those made by Faraday or Darwin. The participatory ideal behind the concept of the Republic of Science from earlier in the century, as noted by Sheets-Pyenson, continued into the 1860s, 1870s and 1880s, shaped by new developments in Victorian publishing, politics, culture and society.

The enduring power of the participatory ideal into the 1880s provokes some interesting reflections on the recent scholarship dealing with the broader issues of professionalization and the creation of scientific icons. As for the former, it has become evident that the attitude of scientific naturalists such as Huxley and Joseph Hooker towards amateurs is more complicated than historians originally thought. Barton has pointed out that we have neglected the significance of amateur members of the X Club, such as John Lubbock. Desmond has shown that when forging alliances, members of the X Club were more concerned with an individual's commitment to naturalism rather than their professional qualifications. In his examination of Hooker's career, Endersby has explored how some scientific naturalists, especially Hooker, distanced themselves from the notion of ‘professional’ as a self-serving interest group with a commercial stake in advancement.^72^ In trying to understand what it meant to be a professional scientist in the second half of the century, at least from the perspective of the scientific naturalists, we cannot simplistically oppose the professional to the amateur. The professionalization of science was a lengthy process. It was far from complete by the end of the century. The scientific naturalists pushing for what they thought of as professionalization were not fanatically opposed to the participation of all amateurs. This may have allowed for the participatory ideal so important to popularizers to survive into the 1880s.

Two of the exemplary scientists celebrated by the popularizers, Faraday and Darwin, have continued to be significant for defining the cultural place of science. But they have not been presented as illustrations of how anyone can participate in the making of science. Near the end of the century, Faraday was appropriated by professional scientists to serve as an icon for the importance of pure science. During the centenary of Faraday's birth in 1891, leading men of science began to recognize the potential of exploiting his reputation in their campaign for more state funding. Faraday could be used to supersede the engineers’ celebration of James Watt as the icon of industrial application of science. Scientists believed that commemorating Watt as the heroic inventor undermined the purely intellectual pursuit of scientific knowledge. Moreover, they were opposed to the emphasis on Watt as an autodidact since it encouraged complacency with regard to scientific and technical education. By 1931, the centenary of Faraday's ‘discovery’ of electromagnetic induction, he had become the personification of scientific modernity, the pure scientist who was responsible for the invention of electrical power, though he too, ironically, can be seen as an autodidact. The Faraday celebrations in 1931 eclipsed the centenary of James Clerk Maxwell's birth and the founding of the British Association for the Advancement of Science.^73^ From the late nineteenth century up to the 1930s, Faraday was preferable to Darwin because of the uncertainty surrounding the fate of the theory of natural selection, as Richmond's account of the 1909 centenary of Darwin's birth illustrates.^74^ It was not until the 1959 celebrations of the centenary of the *Origin of Species* that Darwin became the scientific icon that we are familiar with today, superseding Faraday.^75^ The appropriation of Faraday and, later, of Darwin as iconic figures served to undermine the participatory ideal of the nineteenth-century popularizers and reflected the increasing power of professionalization.

## Notes

1 The *Quarterly Journal of Science* was founded in 1864 and edited by James Samuelson and the chemist William Crookes. Significant changes took place later, first in 1869, when Samuelson became chief editor, and then in 1879, when the journal became a monthly. It ceased publication in 1881. See Ruth Barton, ‘Just before *Nature*: the purposes of science and the purposes of popularization in some English popular science journals of the 1860s’, *Ann. Sci.* **55**, 1–33 (1998), at pp. 6, 18–26.

2 ‘Introduction’, *Q. J. Sci.* **1**, 1–23 (1864), at p. 4.

3 The *Intellectual Observer* was edited by journalist and enthusiastic amateur microscopist, Henry Slack. Reorganized out of *Recreative Science*, the journal began to be published in 1862, surviving until 1871. See Barton, *op. cit.* (note 1), pp. 6–13.

4 ‘The domestication of science’, *Intellect. Obsr* **1**, 471–475 (1862), at pp. 471–472.

5 *Op. cit.* (note 4), p. 473.

6 Susan Sheets-Pyenson, ‘Popular science periodicals in Paris and London: the emergence of a low scientific culture, 1820–1875’, *Ann. Sci.* **42**, 549–572 (1985), at p. 551; Susan Sheets-Pyenson, ‘Low scientific culture in London and Paris, 1820–1875’, PhD thesis, University of Pennsylvania, 1971, pp. 11, 55; Barton, *op. cit.* (note 1), p. 2.

7 Intended to constrain the radical publishers, the ‘taxes on knowledge’ applied to the entire publishing trade. There were taxes on paper (except Bibles), on political content and on advertisements. Before they were reduced, these taxes made cheap publishing for the masses legally risky and economically difficult. See Aileen Fyfe, *Science and salvation: evangelical popular science publishing in Victorian Britain* (University of Chicago Press, 2004), pp. 45–46.

8 James A. Secord, *Victorian sensation: the extraordinary publication, reception, and secret authorship of* Vestiges of the Natural History of Creation (University of Chicago Press, 2000), p. 2.

9 Jonathan R. Topham, ‘Publishing “popular science” in early nineteenth-century Britain’, in *Science in the marketplace: nineteenth-century sites and experiences* (ed. Aileen Fyfe and Bernard Lightman), pp. 135–168 (University of Chicago Press, 2007), at pp. 141–143, 160.

10 Secord, *op. cit.* (note 8), pp. 48–51.

11 Bernard Lightman, *Victorian popularizers of science: designing nature for new audiences* (University of Chicago Press, 2007), pp. 29–30.

12 Lightman, *op. cit.* (note 11), p. 364.

13 Lightman, *op. cit.* (note 11), pp. 12–13.

14 Bernard Lightman, ‘Frank Buckland and the resilience of natural theology’, in *Evolutionary naturalism in Victorian Britain*, pp. 1–27 (Ashgate, Farnham, 2009), at pp. 8–11; Bernard Lightman, ‘Lecturing in the spatial economy of science’, in Fyfe and Lightman (eds), *op. cit.* (note 9), pp. 103–111.

15 Frank Buckland, *Log-book of a fisherman and zoologist* (Chapman & Hall, London, 1875), pp. xi–xii.

16 Buckland, *op. cit*. (note 15), p. vii.

17 Buckland, *op. cit*. (note 15), p. 70. See also Buckland's essay on his conversation with Captain David Gray. Gray's 22 seasons of whaling at the North Pole makes him a reliable source of scientific knowledge. See Frank Buckland, *Notes and jottings from animal life* (Smith, Elder & Co., London, 1882), p. 340.

18 Barton, *op. cit.* (note 1), p. 3; Sheets-Pyenson, ‘Popular science periodicals’, *op. cit.* (note 6), p. 563.

19 The term ‘exemplary scientist’ is not used by the historical actors. It is a term I have coined.

20 J. H. Pepper, *Cyclopaedic science simplified* (Frederick Warne & Company, London, 1869), p. 527.

21 Edited by Shirley Hibberd, *Recreative Science* was a monthly that published two volumes per year. Founded in 1859, it survived until 1871, though under different names and in reorganized formats. It became *The Intellectual Observer* in 1862 and the *Student and Intellectual Observer of Science, Literature and Art* in 1868. See Barton, *op. cit.* (note 1), pp. 6–13.

22 Barton, *op. cit.* (note 1), p. 9; Hain Friswell, ‘Humboldt’, *Recreative Sci.* **1**, 15–20, 55–60 (1860).

23 Sir Robert Ball, *In starry realms* (J. B. Lippincott Company, Philadelphia, 1892), p. 364.

24 Grant Allen, ‘The amateur in science’, *New Sci. Rev.* **1**, 301–308 (1895), at p. 304.

25 Mrs Eliza Brightwen, *Glimpses into plant life: an easy guide to the study of botany* (T. Fisher Unwin, London, 1897), p. 30.

26 Proctor created *Knowledge*, a weekly to begin with, to challenge *Nature* for control of the upscale science periodical market aimed at a popular audience. The format of *Knowledge* reflected Proctor's aversion for the professionalizing, hierarchical vision of science contained in the pages of *Nature*. See Lightman, *op. cit.* (note 11), p. 325.

27 ‘Popular astronomy’, *Knowledge* **1**, 336–337 (February 17, 1882), at p. 336.

28 Richard Proctor, ‘Newton and Darwin’, *Knowledge* **2**, 545–546 (April 28, 1882), at p. 545.

29 Lightman, *op. cit.* (note 11), pp. 302–305.

30 ‘Reviews of books: *Other worlds than ours*’, *Sci. Opin.* **3**, 488 (June 1, 1870).

31 The short-lived *Scientific Opinion* (1868–1870), edited by Henry Lawson, was a weekly known for the quality of its editorial comment. See Barton, *op. cit.* (note 1), pp. 6, 26–27.

32 ‘Mr. Proctor on star-drift and nebulae’, *Sci. Opin.* **3**, 417 (May 11, 1870).

33 ‘Professor Tyndall on haze and dust’, *Sci. Opin.* **3**, 81 (January 26, 1870).

34 ‘Correspondence: Haze and dust. From Dr. Tyndall’, *Sci. Opin.* **3**, 122 (February 3, 1870).

35 ‘The week’, *Sci. Opin.* **3**, 299 (March 30, 1870); ‘The week’, *Sci. Opin.* **3**, 378–381 (April 27, 1870), at p. 378; ‘Dr. Tyndall and the germ theory’, *Sci. Opin.* **3**, 481 (June 1, 1870).

36 Melinda Baldwin, *Making* Nature: *the history of a scientific journal* (University of Chicago Press, 2015), p. 8.

37 [Richard Proctor], ‘The British Association’, *Knowledge* **2**, 257 (September 15, 1882).

38 Richard Proctor, ‘Prize-pig honours for science’, *Knowledge* **9**, 215–216 (May 1, 1886), at p. 215.

39 Proctor, ‘Prize-pig honours for science’, *op. cit.* (note 38), p. 216.

40 Sheets-Pyenson, ‘Popular science periodicals’, *op. cit.* (note 6), p. 563; Barton, *op. cit.* (note 1), p. 3.

41 Bernard Lightman, ‘Huxley and the Devonshire Commission’, in *Victorian scientific naturalism: community, identity, continuity* (ed. Gowan Dawson and Bernard Lightman), pp. 103–130 (University of Chicago Press, 2014), at pp. 113–114.

42 Robert E. Kohler and Jeremy Vetter, ‘The field’, in *A companion to the history of science* (ed. Bernard Lightman), pp. 282–295 (John Wiley & Sons, Chichester, 2016), at p. 282.

43 *Science Gossip*, edited by M. C. Cooke and published by Robert Hardwicke, launched its first issue in 1865, lasting until 1893. It was composed mostly of short pieces on some aspect of natural history.

44 ‘Common things’, *Sci. Gossip* **1**, 217–218 (October 1, 1865), at p. 218.

45 David Page, *Advanced text-book of geology: descriptive and industrial* (William Blackwood and Sons, Edinburgh and London, 1856), p. 6.

46 Brightwen, *op. cit.* (note 25), p. 27; Rev. J. G. Wood, *Out of doors: a selection of original articles on practical natural history* (Longman, Green, and Co., London, 1874), pp. 75, 80.

47 Rev. J. G. Wood, *Common objects of the microscope* (Routledge, Warne, and Routledge, London, 1861), p. 4.

48 Tuffen West, ‘Wonders of a stagnant pool’, *Recreative Sci.* **I**, 154–157 (1860), at p. 155.

49 West, *op. cit.* (note 48), p. 157.

50 Francis T. Buckland, *Curiosities of natural history*, 4th edn (Rudd and Carleton, New York, 1860), p. 17.

51 Buckland, *op. cit.* (note 50), p. 18.

52 Buckland, *op. cit.* (note 15), p. 218.

53 As Inspector of Fisheries, Buckland was required to examine the state of rivers across England and Wales and then submit an annual report. He performed his duties energetically and became a tireless advocate for legislation to control pollution, since this was the first step in restocking the rivers with salmon. Buckland played a crucial role in the preparation of the Freshwater Fisheries Act of 1878. See Lightman, ‘Frank Buckland and the resilience of natural theology’, *op. cit.* (note 14), p. 8.

54 Buckland, *op. cit.* (note 15), p. 216.

55 Pepper, *op. cit.* (note 20), p. 344.

56 Benjamin W. Richardson, ‘On research with the large induction coil of the Royal Polytechnic Institution, with special reference to the cause and phenomena of death by lightning’, *Sci. Opin.* **1**, 543–545 (May 19, 1869); Richardson, ‘On research with the large induction coil of the Royal Polytechnic Institution, with special reference to the cause and phenomena of death by lightning’, *Sci. Opin.* **1**, 563–565 (May 26, 1869).

57 Richardson, *op. cit.* (note 56), p. 543.

58 Rev. J. G. Wood, *Illustrated natural history*, 2 vols, 1, pp. v–vi (Routledge, Warne, and Routledge, London, 1859).

59 Alice Bodington, ‘Micro-organisms as parasites’, *J. Microscopy* **7**, 17–32 (1888), on p. 31.

60 Richard A. Proctor, ‘To our readers’, *Knowledge* **1**, 3 (November 4, 1881).

61 ‘The endeavour’, *Recreative Sci.* **1**, 1–2 (1860), at p. 2.

62 Page, *op. cit.* (note 45), p. 1.

63 W. H. Brock, ‘The development of commercial science journals in Victorian Britain’, in *Development of science publishing in Europe* (ed. A. J. Meadows), pp. 95–122 (Elsevier Science Publishers, Amsterdam, New York, Oxford, 1980), at p. 95.

64 Richard A. Proctor, ‘Gossip’, *Knowledge* **9**, 227–230 (May 1, 1886), at p. 228; Proctor, ‘Gossip’, *Knowledge* **9**, 339–341 (September 1, 1886), at p. 339.

65 *Op. cit.* (note 30).

66 Including a correspondence section in *Knowledge* was not innovative. By the 1880s this was a long-standing practice in many science journals. What set Proctor's *Knowledge* apart was his willingness to give his correspondents more and more space in the journal. However, the correspondence section threatened to overwhelm the rest of the columns in the journal and by 1885 Proctor had to rethink the entire format of *Knowledge*. See Lightman, *op. cit.* (note 11), pp. 341–348.

67 Richard A. Proctor, ‘Gossip’, *Knowledge* **8**, 204–205 (September 4, 1885), at p. 205.

68 [Richard A. Proctor], ‘Science and art gossip’, *Knowledge* **2**, 489 (December 29, 1882).

69 ‘Our past and our future’, *Sci. Opin.* **3**, 1–2 (January 5, 1870), at p. 1.

70 W. T. Stead, ‘My system’, *Cassell's Mag.*, 293–297 (August 1906), at p. 297.

71 ‘Introduction’, *Pop. Sci. Rev.* **1**, 3–7 (1862), at pp. 3–6.

72 Ruth Barton, ‘Huxley, Lubbock and half a dozen others: professionals and gentlemen in the formation of the X Club, 1851–1864’, *Isis* **89**, 410–444 (1998); Adrian Desmond, ‘Redefining the X axis: “professionals”, “amateurs” and the making of mid-Victorian biology: a progress report’, *J. Hist. Biol.* **34**, 3–50 (2001), at pp. 5, 7, 11, 41; Jim Endersby, *Imperial nature: Joseph Hooker and the practices of Victorian science* (University of Chicago Press, 2008), at pp. 22–26.

73 Christine Macleod and Jennifer Tann, ‘From engineer to scientist: reinventing invention in the Watt and Faraday centenaries, 1919–1931’, *Brit. J. Hist. Sci.* **40**, 3, 389–411 (2007), at pp. 392, 394, 404–405, 411.

74 Marsha Richmond, ‘The 1909 Darwin celebration: re-examining evolution in the light of Mendel, mutation and meiosis’, *Isis* **97**, 447–484 (2006).

75 Betty Smocovitis, ‘The 1959 Darwin centennial celebration in America’, *Osiris*, 2nd series, **14**, 274–323 (1999).

